# Simultaneous Acute Appendicitis and Isolated Common Iliac Artery Aneurysm: A Case Report

**DOI:** 10.1002/ccr3.71050

**Published:** 2025-09-28

**Authors:** Meghdad Ghasemi Gorji, Fardin Karbakhsh Ravari, Ali Rafiei, Aisa Iranmanesh, Maral Ebrahimi Alamdari

**Affiliations:** ^1^ Department of Vascular Surgery Shiraz University of Medical Sciences Shiraz Iran; ^2^ Department of Vascular Surgery, Faculty of Medicine Shiraz University of Medical Sciences Shiraz Iran; ^3^ Student Research Committee Shahid Beheshti University of Medical Sciences Tehran Iran

**Keywords:** acute appendicitis, case report, common iliac artery aneurysm, co‐occurrence

## Abstract

This case report presents the rare simultaneous occurrence of acute appendicitis and a large right common iliac artery aneurysm in a 69‐year‐old male with a history of diabetes and hypertension. The patient presented with right lower quadrant abdominal pain and a pulsatile mass. Diagnostic imaging via computed tomography angiography revealed a 95 × 75 mm fusiform aneurysm and radiologic signs of acute appendicitis. Given the high risk of aneurysmal rupture and the presence of symptomatic appendicitis, a combined open surgical approach was undertaken. This involved aneurysm repair with a Dacron graft and standard appendectomy. Histopathology confirmed acute suppurative appendicitis and atherosclerotic changes in the aneurysm wall. The patient recovered well postoperatively, with imaging confirming graft patency and no residual inflammation. This case highlights the importance of comprehensive imaging and a multidisciplinary surgical strategy when managing concurrent intra‐abdominal vascular and gastrointestinal emergencies.

**Summary**

- Concurrent presentation of acute appendicitis and a large iliac artery aneurysm is extremely rare.
- Prompt diagnosis with imaging and a multidisciplinary surgical approach is crucial to prevent rupture and manage infection effectively, highlighting the importance of considering vascular pathology in patients with atypical abdominal findings.

## 1 Introduction

A common iliac artery aneurysm is characterized by a localized enlargement of the artery that exceeds 1.5 cm in diameter [1]. Acute appendicitis is the most prevalent abdominal surgical emergency, though its identification and treatment continue to be challenging for surgeons [2]. The co‐existence of these two medical conditions is highly uncommon and typically indicates a severe clinical situation, requiring timely diagnosis and appropriate intervention [3]. In this report, we describe the successful management of a patient diagnosed with both appendicitis and a common iliac artery aneurysm simultaneously.

## 2 Case History/Examination

We present a case of a 69‐year‐old Iranian male with a medical history of type 2 diabetes mellitus (HbA1c: 7.2%) and hypertension (controlled with amlodipine 10 mg daily), who presented to the surgical emergency department with a two‐day history of progressive, non‐migratory right lower quadrant (RLQ) abdominal pain and low‐grade fever (38°C). The patient denied nausea, vomiting, diarrhea, or urinary symptoms but reported mild anorexia. Physical examination revealed a hemodynamically stable patient (BP: 140/85 mmHg, HR: 88 bpm) with localized tenderness in the RLQ and a palpable, pulsatile mass (approximately 8 cm in diameter) without overlying erythema or warmth. No abdominal bruits, rebound tenderness, or guarding were observed. Peripheral pulses were symmetric and intact.

**FIGURE 1:**
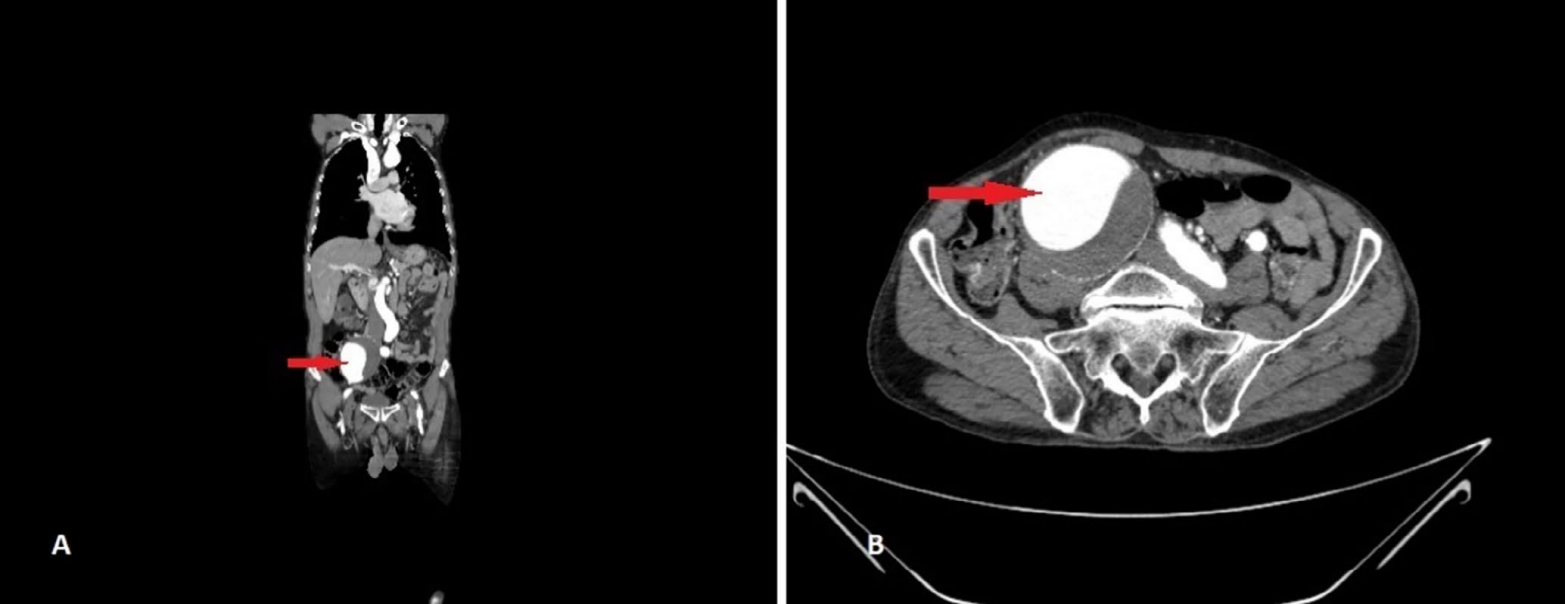
Right common iliac artery aneurysm indicated by a red arrow in coronal (A) and axial (B) views.

**FIGURE 2:**
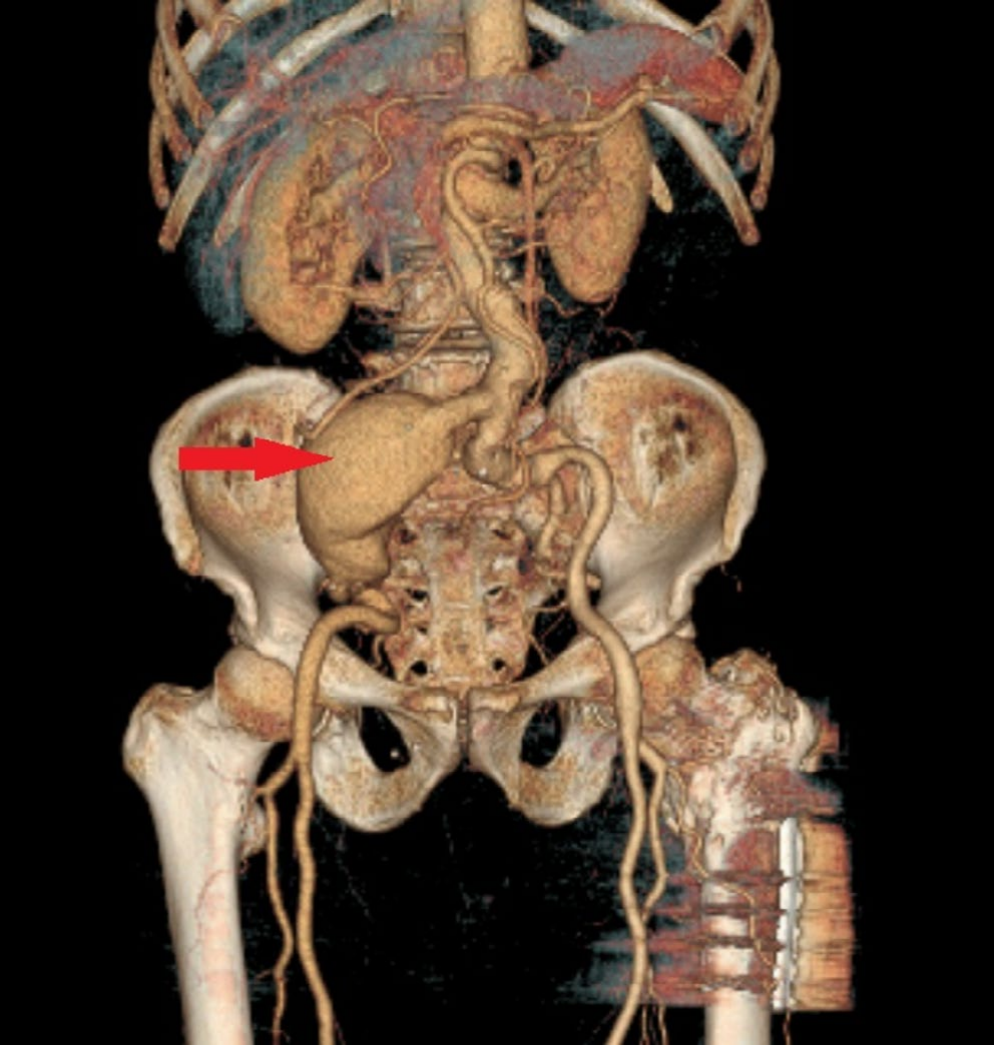
A red arrow indicates the Right common iliac artery aneurysm in a 3D CTA view.

Laboratory evaluation demonstrated leukocytosis (12.5 × 10^9^/L; neutrophils 80%, lymphocytes 15%), elevated C‐reactive protein (35 mg/L; ref.: < 5 mg/L), and normal hemoglobin (14.0 g/dL), platelets (250 × 10^9^/L), renal function (creatinine: 0.9 mg/dL), and liver enzymes (ALT: 25 U/L, AST: 28 U/L) (Table 1). Urinalysis was unremarkable. Given the pulsatile mass and concern for vascular pathology, computed tomography angiography (CTA) was prioritized over ultrasound. CTA revealed a 95 × 75 mm fusiform aneurysm of the right common iliac artery (Figures 1 and 2), originating 2 cm distal to the aortic bifurcation and extending to the iliac bifurcation. The aneurysm exhibited partial intraluminal thrombus, mural calcifications, and mild compression of the adjacent right ureter without hydronephrosis. Notably, the appendix appeared thickened (8 mm diameter) with periappendiceal fat stranding, consistent with acute appendicitis. No aortic or contralateral iliac involvement was observed.

**TABLE 1:** Summary of the patient's biochemical findings upon presentation.

Parameter	Patient value	Reference range	Interpretation
WBC	12.5 × 10^9^/L	4.0–10.0 × 10^9^/L	↑ Leukocytosis
Neutrophils	80%	40%–70%	↑ Neutrophilia
Lymphocytes	15%	20%–40%	↓ Lymphopenia
CRP	35 mg/L	< 5 mg/L	↑ Elevated
Hemoglobin	14.0 g/dL	13.5–17.5 g/dL (men)	Normal
Platelets	250 × 10^9^/L	150–450 × 10^9^/L	Normal
Creatinine	0.9 mg/dL	0.6–1.2 mg/dL	Normal
BUN	14 mg/dL	7–20 mg/dL	Normal
Na^+^	140 mmol/L	135–145 mmol/L	Normal
K^+^	4.2 mmol/L	3.5–5.0 mmol/L	Normal
ALT (SGPT)	25 U/L	7–56 U/L	Normal
AST (SGOT)	28 U/L	10–40 U/L	Normal

## 3 Methods (Differential Diagnosis, Investigations and Treatment)

The patient's presentation posed a diagnostic challenge, as RLQ pain secondary to appendicitis overlapped with symptoms of an expanding iliac aneurysm. Differential diagnoses included appendiceal abscess, diverticulitis, and mycotic aneurysm. However, the absence of gastrointestinal symptoms, coupled with imaging findings, supported concurrent pathologies. The aneurysm's morphology (fusiform, calcified) and lack of perianeurysmal fluid or gas suggested a degenerative etiology, likely atherosclerotic given the patient's age and comorbidities. Serological tests for syphilis (RPR), hepatitis B/C, HIV, and autoimmune markers (ANA, ANCA) were negative, ruling out infectious or vasculitic causes.

**FIGURE 3:**
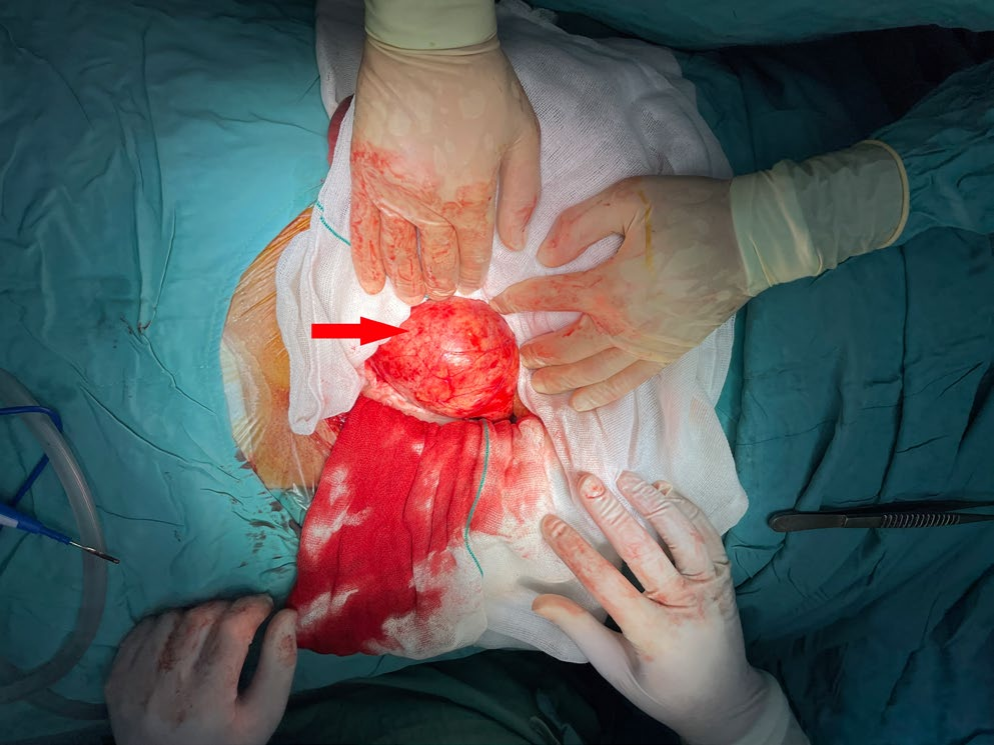
Intraoperative image of the right common iliac artery aneurysm.

**FIGURE 4:**
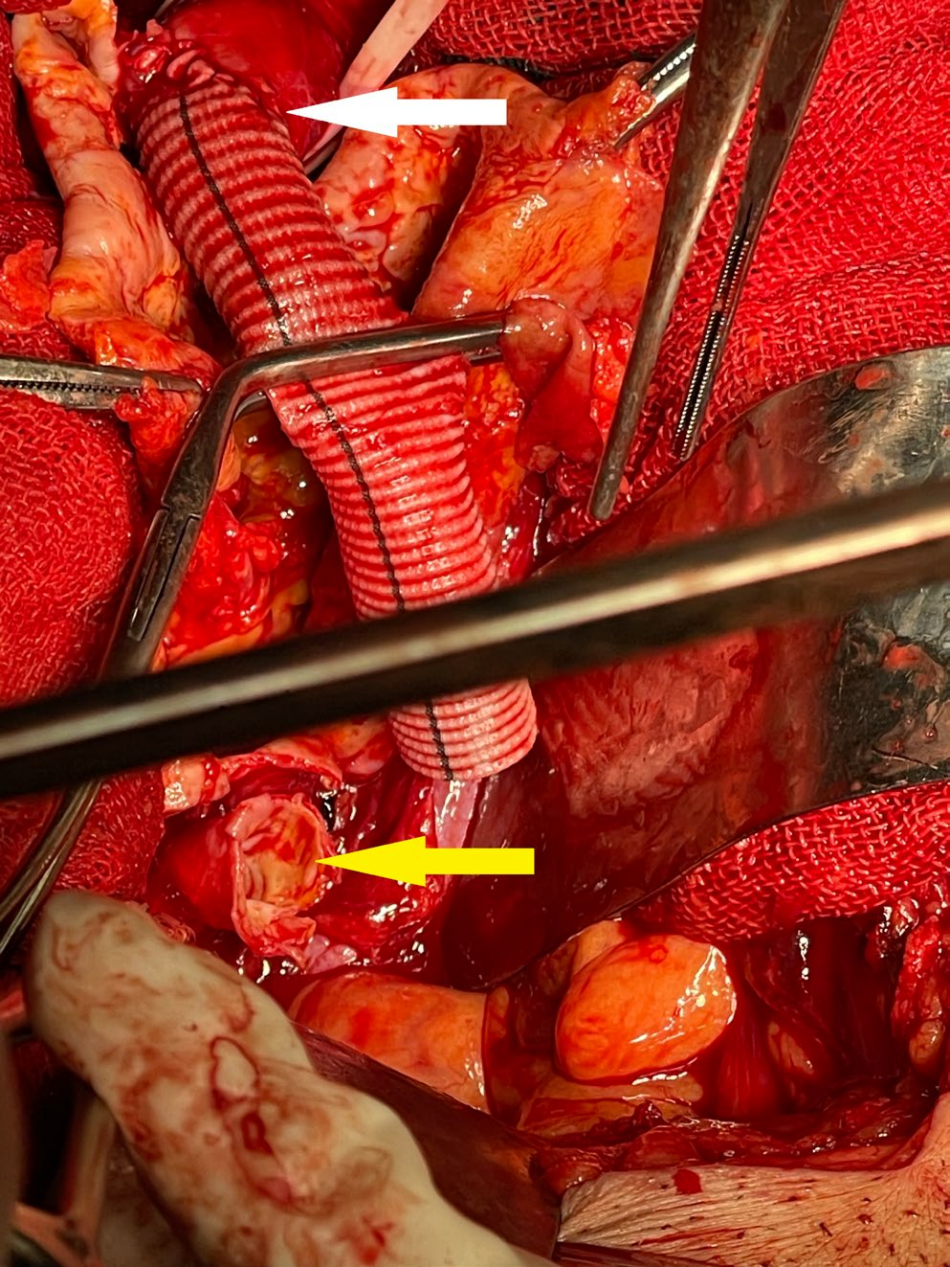
Intraoperative image of right common iliac artery aneurysm repair using a Dacron graft. The white arrow indicates the proximal anastomosis of the graft to the beginning of the right common iliac artery, and the yellow arrow shows the beginning of the right external iliac artery, which was anastomosed distally to the graft.

Given the aneurysm's symptomatic presentation, large size (> 5.5 cm), and rupture risk (estimated annual rupture risk: 10%–15% for iliac aneurysms > 5 cm), alongside acute appendicitis, a combined open surgical approach was deemed necessary. Preoperative echocardiography (ejection fraction: 55%) and cardiology clearance were obtained. Under general anesthesia, a midline laparotomy was performed. Intraoperatively, the aneurysm was adherent to the retroperitoneal structures, including the ipsilateral ureter, which was carefully dissected free (Figure 3). Proximal control was achieved at the infrarenal aorta and distal control at the external iliac artery. The aneurysmal sac was incised, revealing organized thrombus and a degenerated arterial wall. A 16‐mm Dacron graft was interposed between the common and external iliac arteries, with ligation of the right internal iliac artery to prevent type II endoleak (Figure 4). Simultaneously, a macroscopically inflamed appendix (no perforation or gangrene) was identified and resected via standard appendectomy.

## 4 Conclusions and Results (Outcome and Follow‐Up)

**FIGURE 5:**
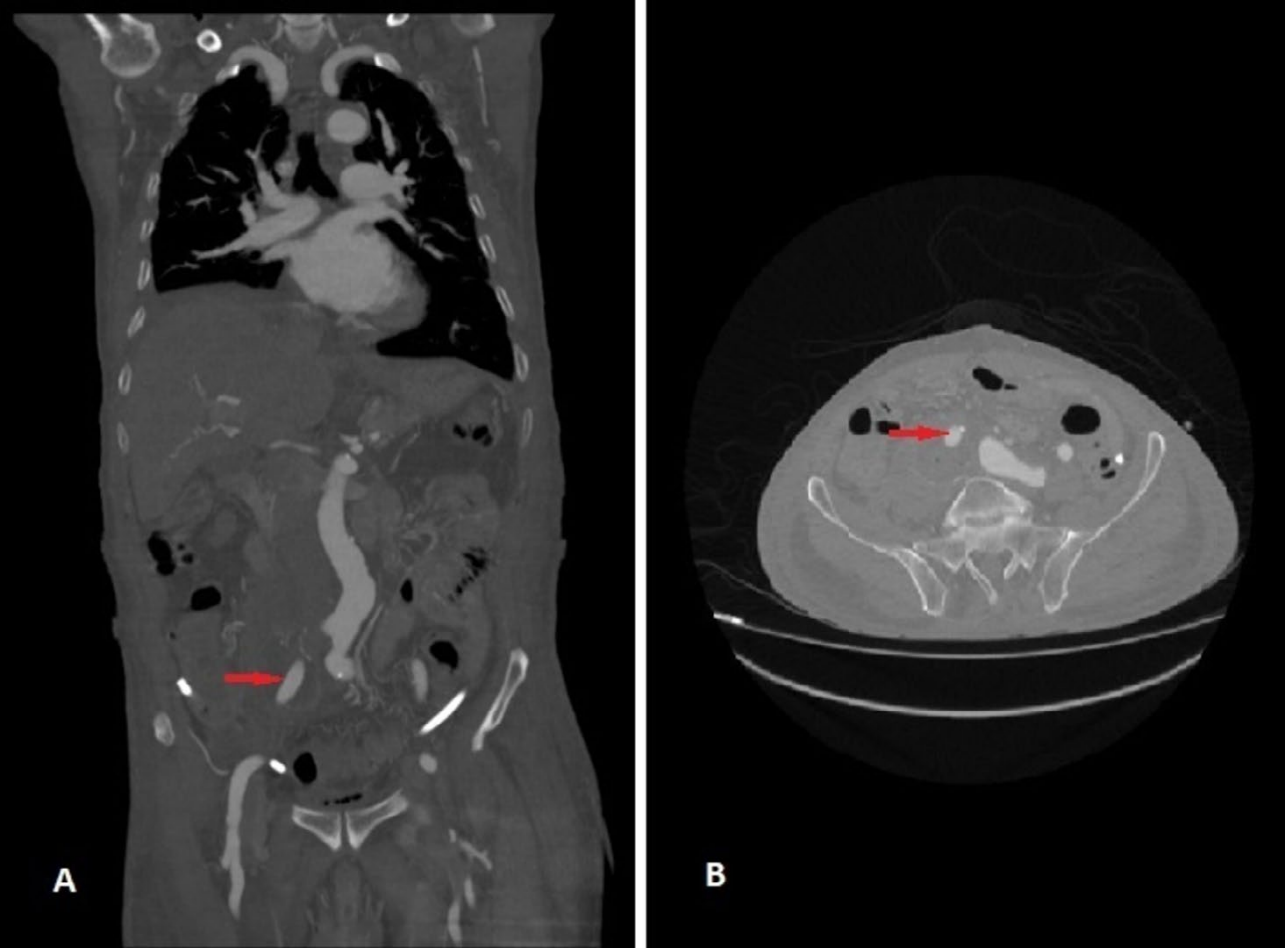
Right common iliac artery aneurysm repaired with a Dacron graft, is shown in the coronal (A) and axial (B) views, indicated by a red arrow that also highlights the graft.

**FIGURE 6:**
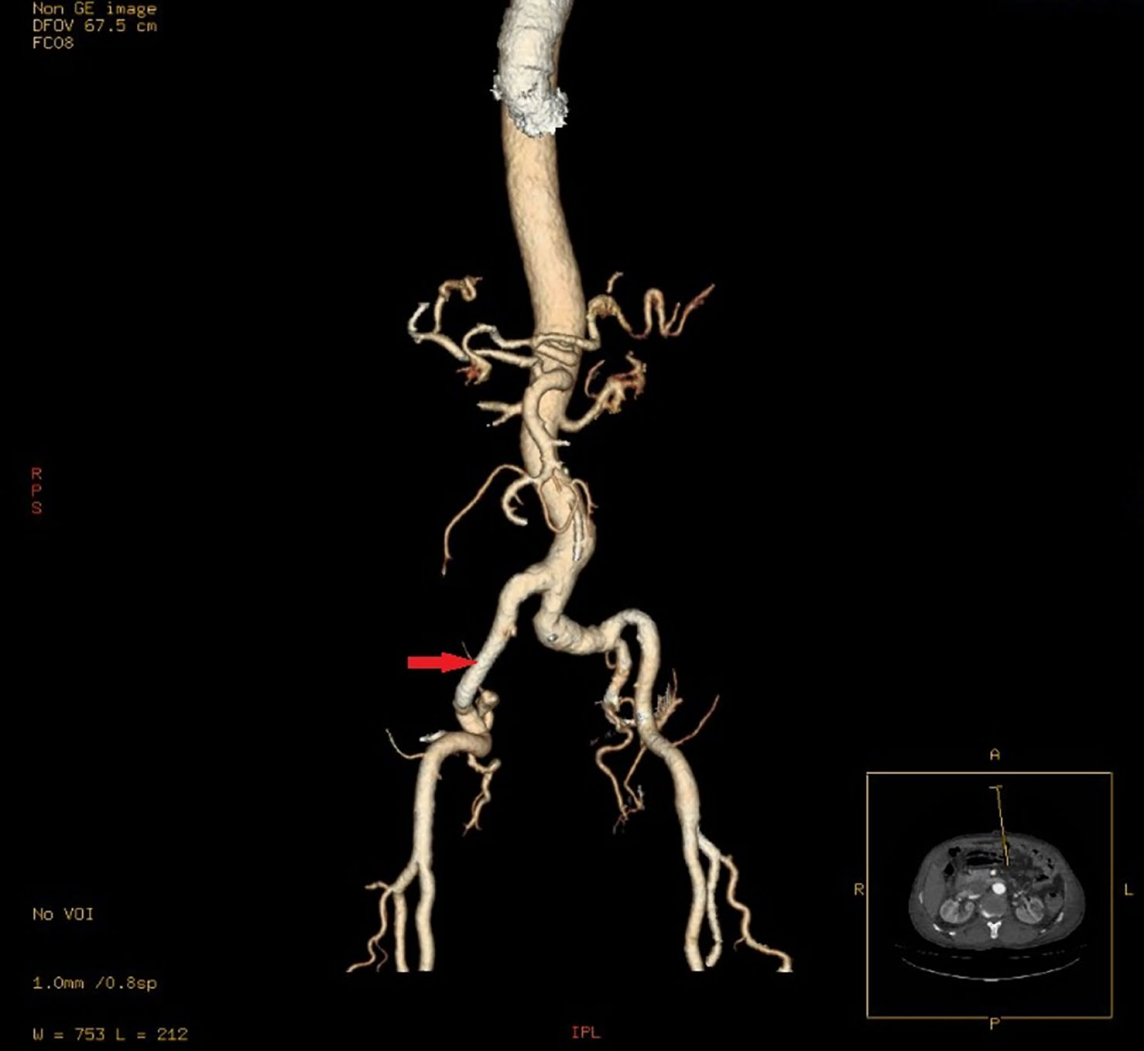
3D CT scan view post repair with a Dacron graft, with the red arrow indicating the graft.

Histopathology of the appendix confirmed acute suppurative appendicitis with transmural neutrophilic infiltration. Aneurysm wall biopsy revealed atherosclerotic changes (foam cells, cholesterol clefts) without evidence of infection or vasculitis. Intraoperative cultures from the aneurysmal thrombus were sterile. Postoperatively, the patient was monitored in the ICU for hemodynamic stability and graft perfusion. Anticoagulation (low‐molecular‐weight heparin) and broad‐spectrum antibiotics (cefazolin) were administered for 48 h. A follow‐up CTA on postoperative day 3 confirmed graft patency, no anastomotic leakage, and resolution of periappendiceal inflammation (Figures 5 and 6). The patient was discharged on postoperative day 7 with scheduled vascular surgery follow‐up.

At two‐week follow‐up, the patient remained asymptomatic, with a healed surgical incision and palpable right femoral pulse. A repeat CTA at 6 weeks demonstrated a stable graft position and no residual aneurysm. Long‐term surveillance (ultrasound at 6 and 12 months) is planned to monitor graft integrity and contralateral iliac vasculature.

This case report has been prepared in accordance with the updated SCARE 2023 guideline, ensuring adherence to standardized principles for surgical case reporting as recommended by Sohrabi et al. [4].

## 5 Discussion

The co‐occurrence of appendicitis and common iliac artery aneurysms is an exceedingly rare clinical scenario, with only a few cases documented in the literature [3]. Appendicitis, a frequent cause of acute abdomen, typically occurs in younger individuals, while common iliac artery aneurysms predominantly affect older adults, particularly those with atherosclerotic disease [5, 6]. The pathophysiologies of these two conditions are distinct: appendicitis generally results from obstruction of the appendiceal lumen, leading to infection and inflammation, whereas common iliac artery aneurysms are frequently associated with risk factors such as atherosclerosis, hypertension, and smoking [5, 6]. This unique co‐occurrence requires a nuanced approach to diagnosis and management, with an emphasis on early recognition and appropriate intervention to avoid potentially life‐threatening complications, such as aneurysm rupture or sepsis.

Diagnosis of both conditions can be challenging, as the symptoms often overlap. Patients may present with right lower quadrant pain, a hallmark of appendicitis, but the pain can also radiate to the back or flank due to the presence of an aneurysm [5, 7]. Imaging techniques, particularly computed tomography (CT) scans, play a pivotal role in the identification of both appendicitis and iliac artery aneurysms, guiding surgical planning. Ultrasound and MRI may serve as adjuncts, particularly in cases where CT imaging is inconclusive or contraindicated [5, 8]. Timely imaging is crucial in distinguishing between the two conditions, as misdiagnosis could result in delayed treatment and worsened outcomes.

Management of co‐occurring appendicitis and common iliac artery aneurysms necessitates a multidisciplinary approach. Appendectomy remains the gold standard for appendicitis, while the treatment of the aneurysm depends on its size, location, and the patient's overall health [5, 9]. Endovascular aneurysm repair (EVAR) is increasingly preferred due to its minimally invasive nature, offering reduced morbidity and quicker recovery times than traditional open surgical repair [10]. However, in cases where EVAR is not feasible, open surgical repair may be required, such as extra‐anatomic bypass or graft placement [10]. Given the complexity of these cases, careful preoperative planning, including antibiotic therapy to mitigate infection, and close collaboration among surgeons, vascular specialists, and anesthesiologists is vital to ensure the best possible outcome for the patient.

The prognosis for patients with both appendicitis and common iliac artery aneurysms depends heavily on the promptness of diagnosis and the effectiveness of surgical intervention. While early intervention can significantly reduce the risk of catastrophic events like aneurysm rupture or systemic infection, the long‐term outcomes remain uncertain, with limited follow‐up data available in the literature [5, 6]. This underscores the need for continued monitoring and follow‐up care to assess the stability of the aneurysm and detect any potential complications, such as graft failure or recurrent infection.

## 6 Conclusion

The simultaneous occurrence of acute appendicitis and a large common iliac artery aneurysm represents an exceptionally rare and potentially life‐threatening clinical scenario. This case highlights the importance of thorough clinical assessment and early use of advanced imaging to identify concurrent pathologies that may present with overlapping symptoms. Successful management requires a tailored, multidisciplinary approach that addresses both conditions in a timely manner, balancing the urgency of infection control with the prevention of aneurysm rupture. Open surgical repair with concomitant appendectomy proved effective in this patient, leading to an uneventful recovery and favorable short‐term outcomes. Long‐term surveillance remains essential to ensure graft integrity and monitor for contralateral vascular changes.

## Author Contributions

**Meghdad Ghasemi Gorji:** conceptualization, investigation, methodology, supervision, validation, visualization, writing – original draft, writing – review and editing. **Fardin Karbakhsh Ravari:** investigation, methodology, validation, visualization, writing – original draft, writing – review and editing. **Ali Rafiei:** investigation, methodology, validation, visualization, writing – original draft, writing – review and editing. **Aisa Iranmanesh:** investigation, writing – review and editing. **Maral Ebrahimi Alamdari:** investigation, methodology, writing – review and editing.

## Ethics Statement

This study followed the ethical guidelines and obtained all necessary approvals.

## Consent

The patient provided written and verbal informed consent, with all necessary explanations.

## Conflicts of Interest

The authors declare no conflicts of interest.

## Data Availability

The data that support the findings of this study are available on request from the corresponding author. The data are not publicly available due to privacy or ethical restrictions.
